# Interactions Between Aging and Alzheimer’s Disease on Structural Brain Networks

**DOI:** 10.3389/fnagi.2021.639795

**Published:** 2021-06-10

**Authors:** Zhanxiong Wu, Yunyuan Gao, Thomas Potter, Julia Benoit, Jian Shen, Paul E. Schulz, Yingchun Zhang, and The Alzheimer’s Disease Neuroimaging Initiative

**Affiliations:** ^1^School of Electronic Information, Hangzhou Dianzi University, Hangzhou, China; ^2^Department of Intelligent Control and Robotics Institute, College of Automation, Hangzhou Dianzi University, Hangzhou, China; ^3^Department of Biomedical Engineering, University of Houston, Houston, TX, United States; ^4^Texas Institute for Measurement Evaluation and Statistics, Department of Basic Vision Sciences, University of Houston, Houston, TX, United States; ^5^Neurosurgery Department, The First Affiliated Hospital of Zhejiang University School of Medicine, Zhejiang University, Hangzhou, China; ^6^Department of Neurology, The McGovern Medical School of UTHealth-Houston, Houston, TX, United States

**Keywords:** Alzheimer’s disease, structural network, nerve fiber tracking, diffusion-weighted magnetic resonance imaging, cognitive impairment

## Abstract

Normative aging and Alzheimer’s disease (AD) propagation alter anatomical connections among brain parcels. However, the interaction between the trajectories of age- and AD-linked alterations in the topology of the structural brain network is not well understood. In this study, diffusion-weighted magnetic resonance imaging (MRI) datasets of 139 subjects from the Alzheimer’s Disease Neuroimaging Initiative (ADNI) database were used to document their structural brain networks. The 139 participants consist of 45 normal controls (NCs), 37 with early mild cognitive impairment (EMCI), 27 with late mild cognitive impairment (LMCI), and 30 AD patients. All subjects were further divided into three subgroups based on their age (56–65, 66–75, and 71–85 years). After the structural connectivity networks were built using anatomically-constrained deterministic tractography, their global and nodal topological properties were estimated, including network efficiency, characteristic path length, transitivity, modularity coefficient, clustering coefficient, and betweenness. Statistical analyses were then performed on these metrics using linear regression, and one- and two-way ANOVA testing to examine group differences and interactions between aging and AD propagation. No significant interactions were found between aging and AD propagation in the global topological metrics (network efficiency, characteristic path length, transitivity, and modularity coefficient). However, nodal metrics (clustering coefficient and betweenness centrality) of some cortical parcels exhibited significant interactions between aging and AD propagation, with affected parcels including left superior temporal, right pars triangularis, and right precentral. The results collectively confirm the age-related deterioration of structural networks in MCI and AD patients, providing novel insight into the cross effects of aging and AD disorder on brain structural networks. Some early symptoms of AD may also be due to age-associated anatomic vulnerability interacting with early anatomic changes associated with AD.

## Introduction

Increasing evidence suggests that both aging and Alzheimer’s disease (AD) can cause deterioration in anatomical brain connections, which is then associated with a decline in cognitive abilities (Peters, 2002; Perl, 2010; Teipel et al., 2016). Normal aging can undermine white matter organization, as nerve fiber loss increases with age. This decrease in the connections between distinct brain parcels contributes to a disruption in the normal flow of information through cortical networks (Betzel et al., 2014; Zhao et al., 2015; Wu et al., 2020). As a neurodegenerative disorder that reduces synaptic transmission (Morabito et al., 2015), AD also causes a gradual breakdown in brain structural connectivity, eventually resulting in dementia (Voevodskaya et al., 2018; Dai et al., 2019; Wu et al., 2019). This disruption of structural connectivity between key functional subregions may ultimately explain the characteristic deficits found in AD patients (Yao et al., 2010; Fischer et al., 2015; delEtoile and Adeli, 2017; Li et al., 2020). These age- and AD-related alterations in white matter organization can profoundly affect topological features of the brain structural network and synergistically damage its integrity (Palop et al., 2006).

Diffusion-weighted imaging (DWI) has often been employed to assess cerebral white matter tracts (Tuch et al., 2003; Sinke et al., 2018; Innocenti et al., 2019; Sotiropoulos and Zalesky, 2019). Pioneering studies have then used graph theory to quantify the brain structural organization, reporting meaningful results on brain networks in normal aging and AD (Yao et al., 2010; Stawarczyk et al., 2012; Ghanbari et al., 2014; Zhao et al., 2015). In particular, alterations in the topology of brain structural networks and their corresponding metrics reflect the regional interactions as they evolve in both normal aging and in AD progression. When used to address normative aging, decreased network efficiency has been demonstrated in hub regions, limiting their capacity to communicate (Gong et al., 2009; Zhao et al., 2015). This is believed to result from degeneration in the white matter microstructure (demyelination, Wallerian degeneration, gliosis, severe fiber loss, etc.; Burzynska et al., 2010; Damoiseaux, 2017; Reishofer et al., 2018) and contributes to lifelong decline (van den Heuvel and Sporns, 2013; Betzel et al., 2014; Gollo et al., 2018). For mild cognitive impairment (MCI) and AD, altered interregional correlations (particularly among the parahippocampal gyrus, medial temporal lobe, cingulum, fusiform, medial frontal lobe, and orbital frontal gyrus; Yao et al., 2010) lead to increased path lengths and decreased network efficiency (Lo et al., 2010; Fischer et al., 2015; delEtoile and Adeli, 2017), suggesting an impairment of structural networks in MCI and AD (He et al., 2008; Daianu et al., 2015; Raj et al., 2015). Especially, a structural *k*-core network analysis (examination of only nodes with a degree of *k* or higher) was performed on normal controls (NCs) and AD patients to investigate brain network breakdown as AD progresses (Daianu et al., 2013). This study found that white matter integrity deteriorated with age and was able to distinguish early MCI-linked white matter alterations from those that occurred during normal aging. The fact that aging and cognitive impairment could separately affect brain networks highlights the unique effects that each has on brain network topology. The interaction of these effects, however, has not yet been thoroughly addressed.

Considering that age effects are not restricted to healthy individuals, it is likely that the age-related disruption of structural networks can exacerbate the cognitive decline in MCI and AD patients. It is, therefore, necessary to recognize the distinct effects of aging and impairment on the brain structural networks, and how these separate factors can interact within both healthy individuals and those with MCI and AD. At the present time, age-related alterations in the structural networks of MCI and AD patients have not been comprehensively explored. In this study, the data from 139 subjects, obtained from Alzheimer’s Disease Neuroimaging Initiative (ADNI) database (Jack et al., 2008) and divided into three age subgroups (56–65, 66–75, and 71–85 years), were used to assess the deterioration of structure that occurs with age. We included 45 NCs, 37 early MCI (EMCI), 27 late MCI (LMCI), and 30 AD patients. Statistical analysis focused on the cross effects between aging and AD progression on the topology of structural connectivity networks and investigated how the global and nodal topological metrics change with age, including network efficiency, characteristic path length, transitivity, modularity coefficient, clustering coefficient, and betweenness. From whole perspective, this study provides a complete view of AD-related topological changes in brain structural connectomes over time.

## Materials and Methods

### Data

**Figure 1:**
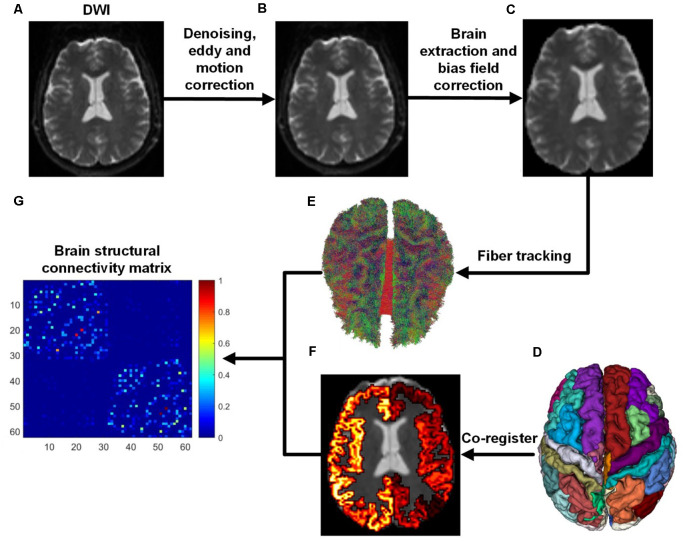
Flowchart of brain structural network construction from diffusion-weighted imaging (DWI). Whole-brain models were parcellated into 62 different parcels according to the DKT template. **(A)** DWI. **(B)** After DWI was denoised, eddy and motion correction were performed. **(C)** Brain extraction and bias field correction. **(D)** DKT parcellation atlas. **(E)** White matter fibers reconstructed with anatomically-constrained tractography (Smith et al., 2012). **(F)** DKT atlas was co-registered into DWI native space. **(G)** Brain structural connectivity matrix was built by assigning fibers to each parcel.

We used the ADNI database (adni.loni.usc.edu), launched in 2003 as a public-private partnership and led by Principal Investigator Michael W. Weiner, MD. The primary goal of ADNI has been to test whether magnetic resonance imaging (MRI), positron emission tomography (PET), biomarkers, and clinical and neuropsychological assessment can be combined to measure the progression of MCI and early AD (Jack et al., 2008; Risacher et al., 2009; Petersen et al., 2010). In this study, 139 subjects aged from 56 to 85 years were selected from the ADNI database, including 45 NCs (32 females and 13 males), 37 EMCIs (18 females and 19 males), 27 LMCIs (11 females and 16 males), and 30 ADs (13 females and 17 males). The criteria for the classification of the subjects was based on mini-mental state examination (MMSE) and global clinical dementia rating (CDR) scores (Aisen et al., 2010). Whole-brain Diffusion-weighted imagings (DWIs) were collected from four MRI centers using the: (1) Siemens 3T scanner (7 b0 images, 48 DWIs with *b* = 1,000 s/mm^2^, slice thickness = 2 mm, scanning sequence = EP, echo time = 0.056 s, repetition time = 7.2 s, flip angle = 90°); (2) the Siemens 3T scanner (13 b0 images, 48 DWIs with *b* = 1,000 s/mm^2^, slice thickness = 2 mm, scanning sequence = EP, echo time = 0.071 s, repetition time = 3.4 s, flip angle = 90°); (3) the GE 3T scanner (6 b0 images, 48 DWIs with *b* = 1,000 s/mm^2^, slice thickness = 2 mm, scanning sequence = EP_SE, echo time = 0.0606 s, repetition time = 7.8 s, flip angle = 90°); and, the (4) Philips 3T scanner (1 b0 image, 32 DWIs with *b* = 1,000 s/mm^2^, slice thickness = 2 mm, scanning sequence = SE, echo time = 0.099 s, repetition time = 10.90 s, flip angle = 90°; Daianu et al., 2013, 2015; Nir et al., 2015). ADNI data collection was performed after obtaining written informed consent from the participants. All procedures were in accordance with the ethical standards of the institutional and/or national research committee and with the 1964 Helsinki Declaration and its later amendments or comparable ethical standards. All DWI images were first denoised and corrected for eddy current and head movement distortions using MRtrix^1^ and FSL^2^ toolboxes. Then, DWI bias field correction was performed by estimating the bias field from b0 images. The whole flowchart of brain structural network construction by DWI is demonstrated in Figure 1.

Of note, there is still considerable controversy in the literature on the statistical analysis of structural network topology from multicenter DWI datasets (Tong et al., 2019). However, we suggest that topological characterization of structural networks would not significantly suffer from multicenter studies, as individual-based analysis of diffusion measures is not sensitive to the variability in MRI scanners (Tong et al., 2020). For the sake of reproducibility, the subject identifiers of each group are provided as Supplementary Materials.

### Structural Network Construction

Brain structural networks can be represented as a graph, completely described by assigning a set of nodes and a set of edges with their corresponding weights (Hagmann et al., 2008; Garcés et al., 2016; Maier-Hein et al., 2017). In order to attain regional anatomical connectivity, the DKT template was applied to parcellate the whole brain into 62 subcortical regions (Potvin et al., 2017). Figure 1D demonstrates the DKT parcellation template, and Table 1 lists the indices of regions of interests (ROIs). This template was co-registered into DWI native space to define ROIs for each subject. The MRtrix tool^1^ was employed to reconstruct fiber tracks using deterministic tractography based on orientation distribution function (ODF) computed with constrained spherical deconvolution (CSD; Tournier et al., 2008). After fiber tracks were retrieved with the command “*tckgen -act*,” spherical-deconvolution informed filtering of tractograms (SIFT) was employed to improve whole-brain streamlines reconstructions with the command “*tcksift*.” Then, an inter-regional anatomical connectivity matrix was then obtained with “*tck2connectome -symmetric -zero_diagonal*,” where the value of any element of the matrix is equal to the number of tracts originating in one region and terminating in (or passing to) another region. The number of fiber tracts between gray matter regions uncovered by MRtrix was determined from the data rather than defined *a priori*, and was therefore variable from individual to individual and from scan to scan (Bassett et al., 2011). Finally, the structural connectivity matrices were normalized into [0, 1] for topological characterization.

**Table 1:** Indexes of ROIs used to construct structural networks (Tzourio-Mazoyer et al., 2002).

Region	Region	Region	Region	Region
**1** Left caudal anterior cingulate	**14** Left parahippocampal	**27** Left superior parietal	**40** Right lateral occipital	**53** Right precentral
**2** Left caudal middle frontal	**15** Left paracentral	**28** Left superior temporal	**41** Right lateral orbitofrontal	**54** Right precuneus
**3** Left cuneus	**16** Left pars opercularis	**29** Left supramarginal	**42** Right lingual	**55** Right rostral anterior cingulate
**4** Left entorhinal	**17** Left pars orbitalis	**30** Left transverse temporal	**43** Right medial orbitofrontal	**56** Right rostral middle frontal
**5** Left fusiform	**18** Left pars triangularis	**31** Left insula	**44** Right middle temporal	**57** Right superior frontal
**6** Left inferior parietal	**19** Left pericalcarine	**32** Right caudal anterior cingulate	**45** Right parahippocampal	**58** Right superior parietal
**7** Left inferior temporal	**20** Left postcentral	**33** Right caudal middle frontal	**46** Right paracentral	**59** Right superior temporal
**8** Left isthmus cingulate	**21** Left posterior cingulate	**34** Right cuneus	**47** Right pars opercularis	**60** Right supramarginal
**9** Left lateral occipital	**22** Left precentral	**35** Right entorhinal	**48** Right pars orbitalis	**61** Right transverse tempora
**10** Left lateral orbitofrontal	**23** Left precuneus	**36** Right fusiform	**49** Right pars triangularis	**62** Right insula
**11** Left lingual	**24** Left rostral anterior cingulate	**37** Right inferior parietal	**50** Right pericalcarine	
**12** Left medial orbitofrontal	**25** Left rostral middle frontal	**38** Right inferior temporal	**51** Right postcentral	
**13** Left middle temporal	**26** Left superior frontal	**39** Right isthmus cingulate	**52** Right posterior cingulate	

### Topological Characterization

To characterize the underlying topological properties of brain structural networks, four commonly-used network-level and two nodal topological metrics were computed for each subject: efficiency, characteristic path length, transitivity, modularity coefficient, clustering coefficient, and betweenness centrality. These metrics were directly retrieved from structural connectivity matrices using the Brain Connectivity Toolbox^3^ in MATLAB (The Mathworks, Inc., Natick, MA, USA; Rubinov and Sporns, 2010).

Network efficiency is a sensitive measure of network alterations that occur in aging and neurodegenerative disorders, which reflects the integration of information transfer within a given network. This effectively characterizes how well the information is communicated within the cerebral cortex and is expected to decrease with age (Gong et al., 2009). This metric is defined as:

(1) $$E_{glob}(G)=\frac{1}{N(N-1)}\sum_{i\neq j\in G}\frac{1}{L_{ij}}$$

where *L*_ij_ is the shortest path length between node *i* and *j* in structural connectivity graph G. *N* denotes the number of nodes in the graph *G*.

The network characteristic path length is the average shortest path length between every pair of nodes in the network, which serves as a measure of overall network integration. This metric is inversely related to network efficiency (Cao et al., 2013) and quantifies the ability for information to be propagated in parallel. This metric was computed as:

(2) $$L(G)=\frac{1}{N(N-1)}\sum_{i\neq j\in G}L_{ij}$$

where *L*_ij_ is defined as the shortest path between node *i* and node *j*.

Transitivity measures the probability that the adjacent vertices of a vertex are connected, which is closely related to the clustering coefficient of a graph, as both measure the relative frequency of triangles (Rubinov and Sporns, 2010).

(3) $$T(G)=\frac{3\lambda (G)}{\tau (G)}$$

where λ(*G*) is the number of triangles in *G*, and τ(*G*) is total number of connected triples of nodes in *G*.

The optimal community structure is a subdivision of the network into nonoverlapping groups of nodes in a way that maximizes the number of within-group edges and minimizes the number of between-group edges. Modularity coefficient is a statistic that quantifies the degree to which the network may be subdivided into such clearly delineated groups. The modularity coefficient is defined as (Rubinov and Sporns, 2010):

(4) $$Q(G)=\frac{1}{2m}\sum_{i,j}\left[w_{i,j}-\frac{k_{i}k_{j}}{2m}\right]\;\delta (c_{i},c_{j})$$

where *w*_i,j_ is the connection weight between node *i* and *j*. *k*_i_ and *k*_j_ are the sums of the weights of the edges attached to nodes *i* and *j*, respectively. *m* is the total link weight in the network overall. δ(*c*_i_, *c*_j_) is 1 when nodes *i* and *j* are assigned to the same module and 0 otherwise. Larger *Q* values are indicative of a highly modular network organization, while lower Q values indicate a more uniform network structure.

To assess the effect of aging and AD progression on local brain regions, node clustering coefficient and betweenness centrality were estimated for each group. The weighted clustering coefficient is the average intensity of all triangles associated with each node, which indicates the extent of local interconnectivity or cliquishness in a network (Daianu et al., 2013; Otte et al., 2015).

(5) $$c_{i}=\frac{2}{k_{i}(k_{i}-1)}\sum_{j,k}{\left(w_{i,j}w_{j,k}w_{k,i}\right)}^{1/3}$$

where *k*_i_ is the degree of node *i*, and *w* denotes the structural connection weight.

Node betweenness centrality is the number of shortest paths that pass through a node (Equation 5). High betweenness centrality values indicate more passages traversing a node. In this work, betweenness centrality was normalized to the range [0, 1] as betweenness/[(*N* − 1)(*N* − 2)] (Rubinov and Sporns, 2010).

(6) $$b_{i}=\sum_{h\neq j,h\neq i,j\neq i}\frac{p_{hj}(i)}{p_{hj}}$$

where *p*_hj_ is the number of shortest paths between nodes *h* and *j*, and *p*_hj_(*i*) is the number of shortest paths between *h* and *j* that pass through the node *i*.

### Statistical Analysis

The objective of this study is to assess the interactive effects of aging and AD progression on topological properties of the brain structural network. After the gender covariate was regressed out, linear regression and ANOVAs were adopted for statistical analysis and performed. In order to estimate the changing trajectories of topological measures over age, linear regression was separately performed on each global metric in the NC, EMCI, LMCI, and AD groups, respectively. To test whether the network-level topology of structural networks was significantly different over age and across NC, EMCI, LMCI, and AD groups, group-wise comparisons of network-level topological measures were performed using one-way ANOVA tests. Finally, to characterize the interaction between aging and AD progression on network-level and nodal topological properties, two-way ANOVA tests with the two factors of age and AD propagation stage were employed to identify group-wise differences. The factor of age consists of three levels: 56–65 years, 66–75 years, and 76–85 years. And the factor of the AD stage includes four levels: NC, EMCI, LMCI, and AD. A significance level of *p*-value <0.05 (uncorrected) was used for ANOVA tests.

## Results

### Linear Regression on Network-Level Topological Metrics

**Figure 2:**
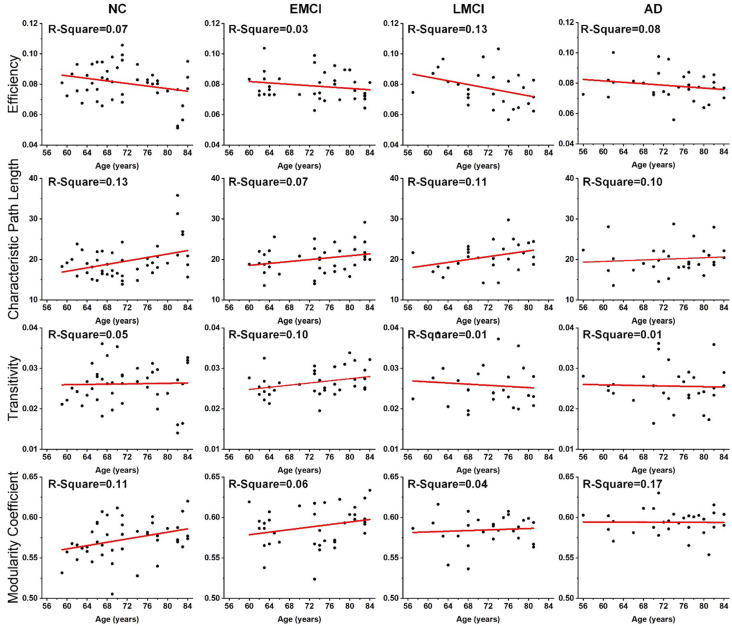
Age effects on the global topological properties of structural networks, including global efficiency, characteristic path length, transitivity, and modularity coefficient. *R*-square value is present on the left corner of each subgraph. The fitted lines are shown in red, and the black dots represent the metric values of each subject.

Linear regression was performed on the global topological metrics (network efficiency, characteristic path length, transitivity, and modularity coefficient) to examine whether, over age, the structural networks of MCI and AD patients exhibited similar deterioration patterns. Figure 2 shows the results, which indicate that the characteristic path lengths (Slope: 0.21, 0.12, 0.18, 0.05) of the NC, EMCI, LMCI, and AD groups increased with age, while the metric of efficiency (Slope: −0.0004, −0.0002, −0.0006, −0.0002) decreased. However, except for the EMCI group, the transitivity of the NC, LMCI, and AD groups were nearly unchanged. While modularity coefficients (Slope: 0.0010, 0.0008, 0.0002) of NC, EMCI, LMCI groups increase with age, the coefficient of the AD group remained unchanged. R-square values of the linear regression were present on the left corner of each subgraph. Overall, linear regression results indicated that the integrity of the structural networks of NC, MCI, and AD individuals all roughly deteriorated with age. However, lesser age-related effects were found in the metrics of the AD group.

### ANOVA Tests on Topological Measures

**Figure 3:**
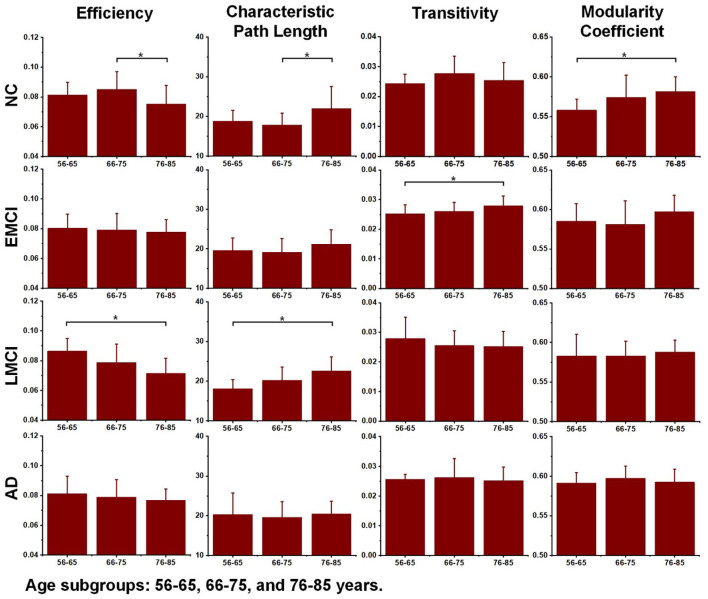
Differences between age subgroups (56–65, 66–75, and 76–85 years) in network efficiency, characteristic path length, transitivity, and modularity coefficient. Group differences were estimated using one-way ANOVA, and the asterisk sign (*) indicates that *p*-value < 0.05 (uncorrected).

Differences in the global topological measures between the three age subgroups (56–65, 66–75, and 71–85 years) were assessed using one-way ANOVA tests. Figure 3 demonstrates the comparison results, and the asterisk sign (*) indicates that *p*-value <0.05 (uncorrected). For the NC group, differences between age subgroups in network efficiency and characteristic path were statistically significant (*p-value* = 0.0116 and *p-value* = 0.0134, respectively). For EMCI, differences in efficiency, characteristic path and clustering coefficient were significant (*p*-value = 0.0467, *p*-value = 0.0256 and *p*-value = 0.0069, respectively). For LMCI subjects, differences between age groups in efficiency and clustering coefficient were significant (*p*-value = 0.0211 and *p*-value = 0.0315, respectively). No metrics significantly differentiated the two age subgroups within the AD group.

**Figure 4:**
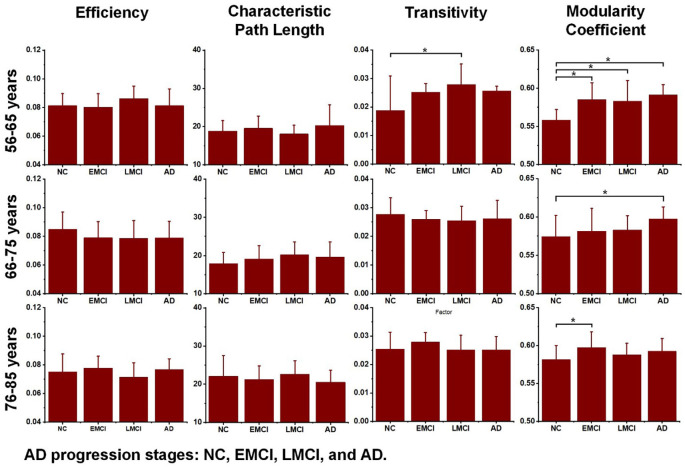
Differences across the normal control (NC), early mild cognitive impairment (EMCI), late mild cognitive impairment (LMCI), and Alzheimer’s disease (AD) groups were estimated using one-way ANOVA tests in terms of network efficiency, characteristic path length, transitivity, and modularity coefficient. The asterisk sign (*) indicates that the *p*-value was < 0.05 (uncorrected).

Additionally, to detect group-wise differences among NC, EMCI, LMCI, and AD subjects, one-way ANOVA tests were also carried out. Results are shown in Figure 4, and pairwise groups that exhibited significant differences were identified and marked with an asterisk sign (*), indicating that *p*-value <0.05 (uncorrected). For the three age groups, efficiency and characteristic paths do not significantly distinguish the NC, EMCI, LMCI, and AD groups. However, for the three age groups, significant differences were only found in the metric of the modularity coefficient. Interestingly, a significant difference between the NCs and LMCI groups was only found in the 56–65 years group. This may be attributed to individual variability. In summary, while the modularity coefficient was most sensitive to AD propagation across 56–75 years, no significant difference was identified in terms of this metric among NC, LMCI, and AD subjects in the 76–85 years group.

**Figure 5:**
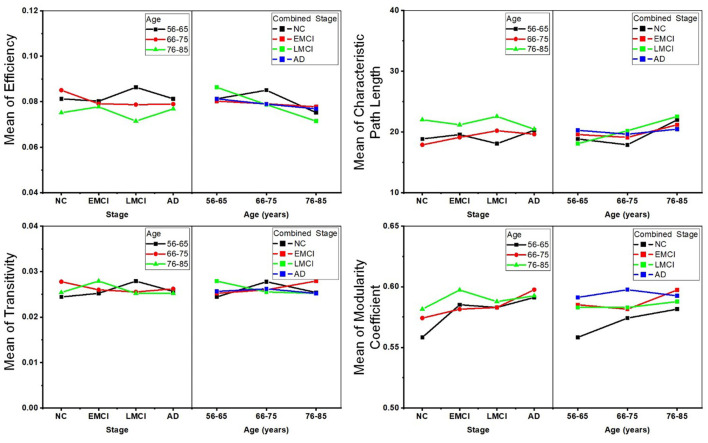
Combined changing trajectories of the four network-level topological metrics (efficiency, characteristic path length, transitivity, and modularity coefficient). No significant interactions were found between aging and AD propagation at the level of *p*-value < 0.05 (uncorrected).

Two-way ANOVA tests were also separately performed on the global topological metrics, and the combined changing trajectories of mean values of these metrics are shown in Figure 5. In summary, no significant interactions were found between aging and AD propagation in terms of these network-level metrics.

**Figure 6:**
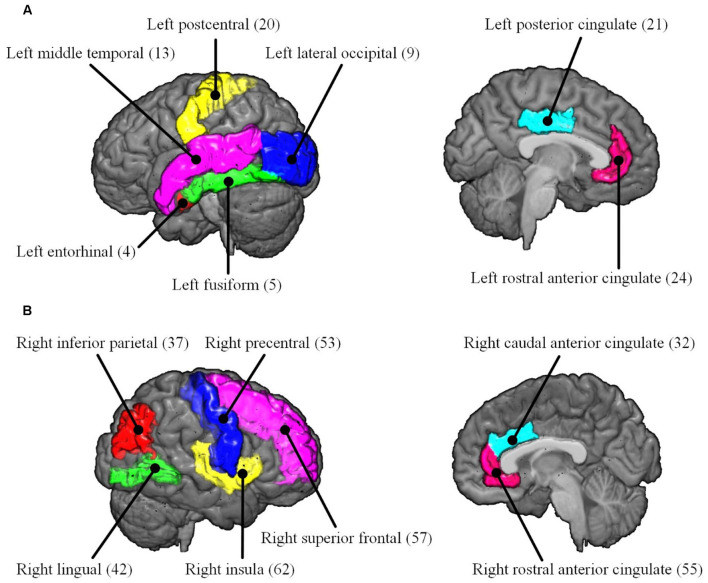
The cortical parcels listed in Table 2 are visualized on MNI152 brain images. The numbers in parentheses are the indexes of parcels (Table 1). **(A)** Left hemisphere. **(B)** Right hemisphere.

**Figure 7:**
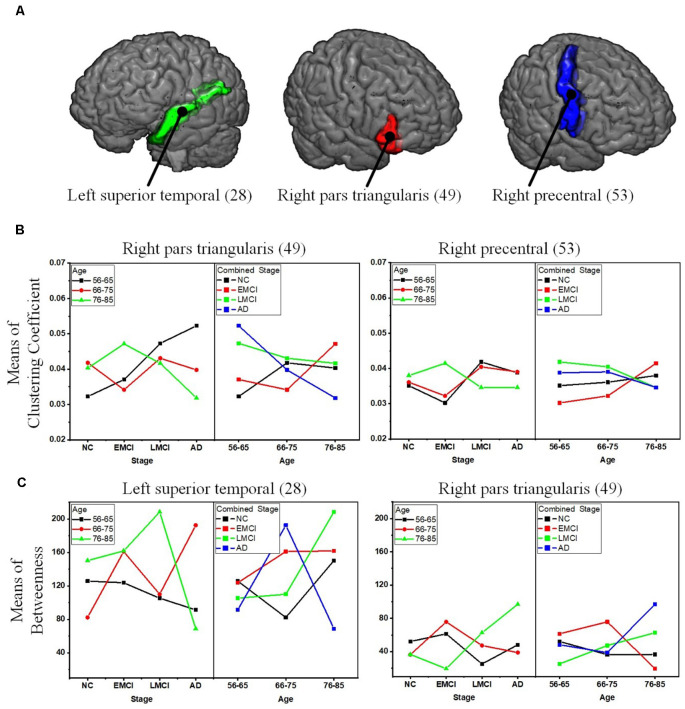
Significant interactions between aging and AD propagation were found in the cortical parcels of left superior temporal, right pars triangularis, and right precentral. **(A)** Visualization of the parcels of left superior temporal, right pars triangularis, and right precentral. **(B)** Combined changing trajectories of mean values of clustering coefficient. **(C)** Combined changing trajectories of mean values of betweenness.

To reveal the interactive effect of aging and AD progression on local topological properties, nodal clustering coefficient and betweenness centrality were estimated for each subject. Using two-way ANOVA tests, it was found that multiple regions, including the left lateral occipital (9), left postcentral (20), right caudal anterior cingulate (32), right inferior parietal (37), right rostral anterior cingulate (55), and right superior frontal (57) exhibited significant differences in terms of clustering coefficient over age (Table 2 and Figure 6). Moreover, significant differences were found only in the parcel of right insula (62) across AD propagation stages age (Table 2 and Figure 6). Regional betweenness centrality values in the left entorhinal (4), left fusiform (5), left middle temporal (13), left posterior cingulate (21), right lingual (42), right precentral (53), right rostral anterior cingulate (55), and right superior frontal (57), showed significant differences across age subgroups (Table 2 and Figure 6). In addition, the left rostral anterior cingulate (24) and right insula (62) parcels were identified to have significant differences across AD stages in terms of betweenness (Table 2 and Figure 6). Finally, for clustering coefficient, the cortical parcels of right pars triangularis (49) and right precentral (53) exhibit significant interaction between aging and AD propagation stages (Table 2 and Figure 7A). For the metric of betweenness, significant interactions between aging and AD stages were found in the left superior temporal (28) and right pars triangularis (49) (Table 2 and Figure 7A). The cortical parcels that exhibited significant groupwise differences and interactions are summarized in Table 2, and the corresponding positions of these parcels are displayed in Figures 6, 7A.

**Table 2:** The cortical subregions with significant differences were identified in terms of clustering coefficient and betweenness centrality.

Nodal metrics	The population means of Age are significantly different (*p* < 0.5)	The population means of Stage are significantly different (*p* < 0.5)	The interaction between Age and Stage is significant (*p* < 0.5)
**Clustering Coefficient**	left lateral occipital (9), left postcentral (20), right caudal anterior cingulate (32), right inferior parietal (37), right rostral anterior cingulate (55), right superior frontal (57)	right insula (62)	right pars triangularis (49), right precentral (53)
**Betweenness**	left entorhinal (4), left fusiform (5), left middle temporal (13), left posterior cingulate (21), right lingual (42), right precentral (53), right rostral anterior cingulate (55), right superior frontal (57)	left rostral anterior cingulate (24), right insula (62)	left superior temporal (28), right pars triangularis (49)

*The listed brain parcels in this table were visualized on Montreal Neurologic Institute 152 (MNI152) brain images, as shown in Figures 6, 7A. The numbers in parentheses were the indexes of cortical parcels (Table 1). The *p*-values were not corrected*.

## Discussion

Cortical connectivity can be seen to reduce with age and AD progression, leading to significant deficits in topological properties of the structural network. These topological metrics provide valuable insights into the deteriorating neurological processes underlying aging and AD progression, offering a unique way to evaluate the impairment of anatomical connectivity patterns. In this study, we constructed brain structural networks of NC, EMCI, LMCI, and AD subjects by calculating fiber bundle numbers between pairs of gray matter parcels and investigating the cross effects of aging and AD progression on network-level and nodal structural topography. The results confirm that normal aging and AD propagation could both affect the integrity of brain structural networks, and indicate that the network-level metrics of AD structural networks were relatively more deteriorated than those of NCs. Overall, however, more significant age-related differences were indicated in healthy controls than in AD patients.

Several recent studies based on DWI have demonstrated that the efficiency of structural networks decreases during normal aging due to neuronal shrinkage, loss of axon fibers, and whiter matter degeneration (Gong et al., 2009; Zhao et al., 2015; Sheffield et al., 2019). To reveal age-related degeneration in the white matter microstructure of NCs, MCI, and AD, this study performed linear regression on each of the global topological metrics, separately. Results provided new insight into the age-related changes in brain structural networks of healthy, MCI, and AD individuals, which are crucial for understanding how age affects the structural connectome of AD disorders. For all groups, network efficiency decreased with increasing age while characteristic path length increased. This is in accordance with previous studies (Meunier et al., 2009; Betzel et al., 2014; Fischer et al., 2015; Zhao et al., 2015). As shown in Figure 2, the deteriorated network-level topological properties of brain structural networks found in this study may provide the underlying substrate for the functional decline observed in aging individuals. In terms of the metrics of efficiency, characteristic path length, and modularity coefficient, the age-related deterioration in structural networks of AD patients is less significant than for healthy and older adults with MCI. We may infer that the anatomical connectivity breakdown caused by AD weakens the detrimental effect of aging on brain structural networks. Additionally, no significant age-related differences were identified in the AD subgroup (Figure 3), weighing against the hypothesis that aging leads to a vulnerability to the spread of AD.

AD progression can be characterized by a loss of connected areas in terms of global topological measures including network efficiency, characteristic path length, transitivity, and modularity coefficient. Much evidence from previous studies supports the interpretation of AD as a disconnection syndrome (Fischer et al., 2015; Morabito et al., 2015; Guo et al., 2016). To further reveal how AD propagation affects structural networks, one-way ANOVA tests were employed to explore groupwise differences across the NC, EMCI, LMCI, and AD groups in each age subgroup (56–65, 66–75, and 76–85 years). Figure 4 demonstrates that in the 56–65- and 66–75-years age groups, there were significant differences between NCs and ADs in terms of modularity coefficient. For the 75–85 years group, however, no significant difference in modularity coefficient was detected, indicating that aging and AD both lead to inter-module disconnection in brain structural networks. Interestingly, as shown in Figure 4, the other global metrics (network efficiency, characteristic path length, and transitivity) did not exhibit significant differences between NC and AD subjects in the three age groups (*p*-value > 0.05). The reasons why there is no difference between the groups regarding efficiency and path length are manifold. This work is based on cross-sectional ADNI data. There are individual variabilities in brain structural networks. The progression stages of AD are generally defined by MMSE and CDR test scores. At present, it’s unclear if the subjects with the same test score share the same brain structural networks. Another hypothesis could be that neural plasticity would alter structural connectivity during AD progression. Some subjects may have better neural plasticity than others. Hence, their connectivity could be better or worse than predicted. These one-way ANOVA tests indicate that the AD subjects are associated with greater structural connectivity deterioration in younger adults, while cognitive impairments have relatively lesser effects on older adults. Age-related alterations of whole-brain white matter network properties of AD patients were not detectable. However, the underlying neurophysiological reasons may be worthy of further study.

To comprehensively reveal the interaction between aging and AD progression on brain structural networks, using two-way ANOVA tests, the cross effect of aging and AD progression on local topological properties has been assessed in terms of node clustering coefficients and betweenness centrality (Table 2, Figures 6, 7). The results indicate that aging and AD progression interactively and significantly affect some local regions, including the left superior temporal, right pars triangularis, and right precentral. This may occur due to broken anatomical connections between these cortical subregions and others, which were interactively affected by aging and AD progression. The three parcels are related to language understanding and motor movement (Foundas et al., 1996; Yousry et al., 1997; Aeby et al., 2013), and these cognitive functions both gradually deteriorate with age and AD progression. Previous studies have found that subjects with MCI and AD have a significant reduction in structural connectivity in the superior temporal lobe, medial temporal lobe, inferior parietal areas, and lingual gyri (Bell-McGinty et al., 2005; Yao et al., 2010; Zhao et al., 2015). Age-related structural network studies also revealed that regional efficiency reduced in the parietal and occipital lobes with age (Gong et al., 2009; Burzynska et al., 2010; Zhao et al., 2015). To some extent, our result is in accordance with these prior studies. Specifically, no significant group-wise difference or interaction was found in the occipital area and hippocampus subregions (14 and 45) in this study. A possible reason for this may be the choices of whole-brain parcellation atlas and nodal metrics. Additionally, as the most serious hippocampal pathology may be already present when the diagnosis of MCI or AD was made, hippocampal connections could not have much additional deteriorations over time. The present results are, to some extent, consistent with these studies: cognitive function deficits could be due to abnormalities in the connectivity between these brain areas. This region-specific topological analysis provides insight into the aberrant topological patterns induced by interaction between aging and AD propagation.

Several methodological issues about this study should be addressed. First, the DKT atlas was used to parcellate the whole cortex. When different parcellation schemes are used to define network nodes, topological metrics may be different (Wu et al., 2019). Second, the edges of the white matter networks were reconstructed by deterministic tractography based on CSD. Future studies should employ more advanced tractography techniques, such as probabilistic tractography to define the network edge weights (Sotiropoulos and Zalesky, 2019). Third, to ascertain the real structural networks as accurately as possible, this study included as many subjects as were available from each group in the ADNI database, which made the sample sizes of each group inconsistent. Fourth, as the DWI datasets are collected from multiple MRI centers, network consistency still needs to be confirmed. For different patients, AD onsets may start in distinct brain areas (Ossenkoppele et al., 2020), and this may influence the statistical analysis of local topological characterization. Finally, as the cause for white matter hyperintensities remain uncertain (Merio, 2019), we did not consider this factor in the statistical tests. Interaction across aging, AD progression, and neural plasticity (Bernhardi et al., 2017) complicates the analysis of brain structural connectivity deterioration due to AD. In the future, the combination of the multimodal MRI techniques (structural, diffusion-weighted, and functional MRI) should yield a comprehensive understanding of the relationship between structural and functional changes during normal aging and AD progression.

## Conclusion

Brain network analysis offers a promising new approach to track and understand aging and AD progression. From this study, we conclude that age-related deterioration in structural networks contributes less to AD patients than healthy old adults. While no significant interaction is identified between aging and AD propagation in terms of the network-level metrics, significant interaction is found in the parcels of left superior temporal, right pars triangularis, and right precentral in terms of nodal clustering coefficient and betweenness. These findings may explain how network abnormalities in AD patients gradually evolve over time. In summary, our results emphasize age- and AD-related degeneration of specific brain parcels, thus providing novel insights into the underlying pathophysiological mechanisms of connectivity alterations over aging and AD progression. This also indicates the potential of using these parcels’ topological metrics as a diagnostic biomarker. Further studies for neurophysiological correlation between aging and AD progress are still needed to comprehensively assess their cross effects on the integrity of structural connectivity.

## Data Availability Statement

The original contributions presented in the study are included in the article, further inquiries can be directed to the corresponding author.

## Ethics Statement

Ethical review and approval was not required for the study on human participants in accordance with the local legislation and institutional requirements. Written informed consent for participation was not required for this study in accordance with the national legislation and the institutional requirements.

## Author Contributions

ZW analyzed and interpreted the data and was a major contributor in writing the manuscript. YG assisted in analyzing data and interpreting the results, and also contributed to manuscript writing. TP assisted in interpretation of the results and manuscript writing. JB performed the statistical analysis and assisted in manuscript writing. JS assisted in analyzing and interpreting the data, and also contributed to manuscript writing. PS assisted in the result interpretation and manuscript writing and revision. YZ supervised the design of study, interpreted results, and contributed to manuscript writing. All authors contributed to the article and approved the submitted version.

## Conflict of Interest

The authors declare that the research was conducted in the absence of any commercial or financial relationships that could be construed as a potential conflict of interest.

## Footnotes

^1^https://www.mrtrix.org

^2^https://fsl.fmrib.ox.ac.uk/

^3^http://www.brain-connectivity-toolbox.net/
